# Prokaryotic Expression, Purification, and Antibacterial Activity of the Hepcidin Peptide of Crescent Sweetlips (*Plectorhinchus cinctus*)

**DOI:** 10.3390/cimb45090456

**Published:** 2023-08-31

**Authors:** Peixin Wang, Zhongjing Lin, Shaoling Lin, Baodong Zheng, Yi Zhang, Jiamiao Hu

**Affiliations:** 1 Engineering Research Centre of Fujian-Taiwan Special Marine Food Processing and Nutrition, Ministry of Education, Fuzhou 350002, Chinashaoling.lin@fafu.edu.cn (S.L.); zbdfst@163.com (B.Z.); 2 College of Food Science, Fujian Agriculture and Forestry University, Fuzhou 350002, China; 3 College of Life Sciences, University of Leicester, Leicester LE1 7RH, UK

**Keywords:** crescent sweetlips, hepcidin, soluble expression, optimization, antibacterial activity

## Abstract

The hepcidin peptide of crescent sweetlips (*Plectorhinchus cinctus*) is a cysteine-rich, cationic antimicrobial peptide that plays a crucial role in the innate immune system’s defense against invading microbes. The aim of this study was to identify the optimal parameters for prokaryotic expression and purification of this hepcidin peptide and characterize its antibacterial activity. The recombinant hepcidin peptides were expressed in *Escherichia coli* strain Arctic Express (DE3), with culture and induction conditions optimized using response surface methodology (RSM). The obtained hepcidin peptides were then purified before tag cleavage, and their antibacterial activity was determined. The obtained results revealed that induction temperature had the most significant impact on the production of soluble recombinant peptides. The optimum induction conditions were determined to be an isopropylthio-β-galactoside (IPTG) concentration of 0.21 mmol/L, induction temperature of 18.81 °C, and an induction time of 16.01 h. Subsequently, the recombinant hepcidin peptide was successfully purified using Ni-IDA affinity chromatography followed by SUMO protease cleavage. The obtained hepcidin peptide (without His-SUMO tag) demonstrated strong antimicrobial activity in vitro against *V. parahaemolyticus*, *E. coli*, and *S. aureus*. The results showed prokaryotic (*E. coli*) expression is a feasible way to produce the hepcidin peptide of crescent sweetlips in a cost-effective way, which has great potential to be used as an antimicrobial agent in aquaculture.

## 1. Introduction

In recent years, numerous studies have suggested that antimicrobial peptides have a broad spectrum of actions against microorganisms, including bacteria, fungi, and viruses, and could be used as alternatives to antibiotics to overcome antibiotic resistance [1,2]. Antimicrobial peptides have been identified in many organisms and play important roles in innate immunity against invading microorganisms [3]. Hepcidin, a cysteine-rich cationic antimicrobial peptide, was acknowledged for its antimicrobial and iron regulation activities [4]. Mammalian hepcidin is known to possess antimicrobial activity and play an important role in regulating the absorption, transport, storage, and mobilization of iron [5]. Meanwhile, the fish hepcidin genes in the liver and other tissues could be induced by bacterial infection and inflammation, indicating that their biological function is mainly related to immunity [6]. For instance, hepcidin genes have been identified in the livers of various fish, such as *Salmo trutta* [4], *Oreochromis niloticus* [7], and *Boleophthalmus pectinirostris* [8]. In our previous work, we successfully identified the hepcidin peptides from crescent sweetlips (*Plectorhinchus cinctus*) (GenBank: UHK02597.1). However, the costs of obtaining hepcidin through extraction or chemical synthesis were far beyond its economic benefits in aquaculture or the food industry. Therefore, finding a cost-effective way to produce antibacterial peptides (e.g., hepcidin) has hitherto received a great deal of attention.

To date, prokaryotic expression systems have become the most promising strategy to produce antibacterial peptides in an economical way. Particularly, the *E. coli* expression system is considered to be the least expensive and most effective protein expression system, with the advantages of rapid bacterial growth, quick expression, and high product yields [9]. For instance, it has been reported that the cost of producing antimicrobial peptide GKY20 using the *E. coli* expression system was 42 €/mg when the production scale was 1000 mg/batch, which was highly competitive when compared to chemical synthesis strategies (260 €/mg). This cost could be further decreased with the expansion of production scale [10].

Notably, enhancement of the solubility of recombinant antibacterial peptides is considered crucial for increasing their production yield and activity [11]. Indeed, it has been reported that more than 30% of recombinant proteins expressed in the *E. coli* expression system appear to form insoluble inclusion bodies [9]. Inclusion bodies are usually made up of aggregated proteins that are misfolded and thus biologically inactive [12]. Indeed, previous studies have illustrated that the prokaryotic expression of fish hepcidin using the *E. coli* expression system is challenging. As a cysteine-rich peptide, the intramolecular disulfide bonds between these cysteine residues of hepcidin peptides have been found to be crucial for its correct folding and bioactivity. However, due to the physiological features of prokaryotic bacteria, the expression of peptides with complex disulfide bridges has been considered difficult in *E. coli* [13]. Furthermore, antibacterial peptides are often very short; therefore, their correct folding is often interfered with by expression tags [14]. For example, Qu et al. (2013) found that *Epinephelus coioides* hepcidin failed to be expressed in *E. coli* BL21 (DE3) pLysS cells [15]. Similarly, Tao et al. (2014) reported that the trxA-channel catfish hepcidin fusion protein possessed no anti-bacterial activity, which required enterokinase to remove the chaperonin [16]. Recently, the His-SUMO tag has become a widely used expression tag for the recombinant expression of antimicrobial peptides. The SUMO tag can significantly enhance the solubility and yield of the recombinant protein, while the His tag is beneficial for its purification. Furthermore, the His-SUMO tag could be easily removed by SUMO protease, which will facilitate the generation of the target peptide with its native N-terminus.

The optimization of induction conditions, such as isopropylthio-β-galactoside (IPTG) concentration, induction time, and induction temperature, has also been proven to greatly enhance the solubility of recombinant peptides produced by prokaryotic expression systems [17]. For example, Papaneophytou & Kontopidis (2016) applied RSM for the optimization of induction conditions of recombinant HO-1 (cell density before induction, IPTG concentration, and post-induction temperature), and a 91% increase in soluble protein production was achieved [18]. Tarahomjoo et al. (2022) reported that optimizing the culture conditions (expression temperature, inducer concentration, and sorbitol inclusion in the culture medium) by RSM could increase the proportion of soluble recombinant CRM197 production from 15.4% to 96.5% [19]. Moreover, the successful optimization of induction temperature, induction time, cell density, and inducer concentration for soluble scFv utilizing RSM was reported by Behravan & Hashemi (2021) [20].

In the present study, the crescent sweetlips hepcidin gene was fused to a His-SUMO gene, and the fusion peptide was efficiently expressed using the *E. coli* Arctic Express (DE3) strain. Then RSM based on a Box-Behnken design was used to optimize the parameters of induction conditions, including IPTG concentration, induction time, and induction temperature. The recombinant hepcidin antibacterial peptide was further purified by Ni-IDA affinity chromatography with the removal of the SUMO tag using SUMO protease. The antibacterial activity of the obtained hepcidin peptide was determined and compared with that of chemically synthesized hepcidin peptides.

## 2. Materials and Methods

### 2.1. Materials

pSUMO-Mut vector and *Escherichia coli* Arctic Express (DE3) were purchased from Zoonbio Biotechnology Co., Ltd. (Nanjing, China). IPTG was purchased from Sigma-Aldrich Co. (St. Louis, MO, USA). LB, TB, SOC, SOB, and 2× YT mediums were purchased from Beijing Solarbio Science & Technology Co., Ltd. (Beijing, China). *Vibrio parahaemolyticus* (ATCC 17802) was obtained from the American Type Culture Collection (ATCC, Rockville, MD, USA). *Escherichia coli* O157:H7 (CICC 25013) and *Staphylococcus aureus* (CICC 10201) were obtained from the China Center of Industrial Culture Collection (CICC, Beijing, China). All other chemicals and reagents were of analytical grade.

### 2.2. Plasmid Constructs

The complete open reading frame of the crescent sweetlips hepcidin gene, containing 273 bp, was obtained according to our previous study (GenBank accession number: MZ773641.1). The hepcidin gene encoded an antimicrobial peptide with 90 amino acids (GenBank accession number: UHK02597.1), and its secondary structures were predicted to be 34.44% α-helix, 16.67% β-sheet, 4.44% β-turn, and 44.44% random coil (https://npsa-pbil.ibcp.fr/cgi-bin/npsa_automat.pl?page=npsa_sopma.html (accessed on 17 August 2023)). The DNA fragment containing the nucleotide sequence of mature crescent sweetlips hepcidin peptide was inserted into the pSUMO-Mut vector in a seamless cloning way (XhoI site). The recombinant plasmid pSUMO-Mut-hepcidin, confirmed by DNA sequencing, was then transformed into *Escherichia coli* Arctic Express (DE3).

### 2.3. Optimization of Induction Conditions

A single isolated colony was inoculated in 3 mL of LB liquid medium (containing 50 μg/mL kanamycin) and grown at 37 °C for 8 h in a shaker incubator at 220 r/min. Then, the bacterial suspension was made in 30 mL of different culture mediums (LB, TB, SOC, SOB, and 2× YT) containing kanamycin (50 μg/mL) and incubated at 37 °C until the mid-exponential growth phase was achieved (OD_600nm_ = 0.8). The bacterial suspension was centrifuged at 10,000 r/min for 2 min at room temperature. Then, the bacteria were resuspended in 100 μL 1× loading buffer. After inducing with 0.2 mM IPTG for 4 h at 15 °C and 220 r/min, 1 mL of culture was centrifuged at 10,000 r/min for 2 min and resuspended in 100 μL 1× loading buffer. The remaining cultures were centrifuged at 4000 r/min for 10 min and resuspended in PBS. After being broken by an ultrasonic wave, the supernatant and precipitate were added to the loading buffer. The cultures (un-induced culture, induced culture, supernatant of the bacteria lysate, and sediment of the bacteria lysate) were analyzed on 12% SDS-PAGE. The medium with the highest expression was selected for the next experiment.

To increase the yield of soluble fusion peptide, a variety of independent cultivation parameters such as IPTG concentration (0.1, 0.2, 0.3, 0.4, and 0.5 mM), induction time (8, 12, 16, 20, 24 h), and induction temperature (15, 20, 25, 30, 35 °C) were optimized. The fusion peptide was quantified by SDS-PAGE and ImageJ software (version 1.53e, National Institutes of Health, Rockville, MD, USA).

### 2.4. RSM

To systematically evaluate the effects of three independent variables, including IPTG concentration (factor A), induction time (factor B), and induction temperature (factor C), on the production of His-SUMO-hepcidin in *E. coli*, an experimental design was developed using the Box-Behnken factorial design scheme. Each variable was investigated at three levels (Table S1), which produced 17 tests in total. When the 17 tests were completed, samples were analyzed by 15% SDS-PAGE, and the yield of the target peptide was assessed by ImageJ software (version 1.53e). The experimental results were then used for RSM analysis by Design Expert software (version 8.0.6, Stat-Ease Inc., Minneapolis, MN, USA).

### 2.5. Purification of Recombinant Peptide

Under the optimized conditions, the recombinant strain was induced to express His-SUMO-hepcidin. After being broken by an ultrasonic wave, the supernatant was passed through a 0.45 μm microfiltration membrane to remove particulates, and then it was purified using an Akta Purifier 10 purification system (GE Healthcare, Chicago, IL, USA) equipped with a Ni-IDA-Sepharose CL-6B affinity chromatography column. The sample was loaded onto the column, which was pre-equilibrated with Ni-IDA binding buffer at 0.5 mL/min. After that, the column was washed by Ni-IDA Washing Buffer (20 mM Tris-HCl, 30 mM imidazole, 150 mM NaCl, pH 8.0) at a flow rate of 1 mL/min until the absorbance value at 280 nm of the eluate reached baseline. Then, the peptide was eluted from the column with a Ni-IDA washing buffer containing 20 mM Tris-HCl, 250 mM imidazole, and 150 mM NaCl (pH 8.0) at a flow rate of 1 mL/min. The collected peptide solution was dialyzed in PBS buffer for 12 h. The peptide solution was analyzed by 12% SDS-PAGE.

### 2.6. Western Blot Analysis

Western blotting was performed according to the method of X. Zhou et al. (2021) [21]. The purified peptide was separated by SDS-PAGE and transferred to a polyvinylidene difluoride (PVDF) membrane. The membrane was washed with PBS-Tween 20 (PBST, 0.1% Tween 20) buffer for four times and incubated with anti-6×His tag mouse monoclonal antibody (Zoonbio Biotechnology, Nanjing, China) diluted by 5% skim milk blocking buffer (1:1000) at 4 °C overnight. After washing with PBST buffer three times, the membrane was incubated with a second antibody (goat anti-mouse IgG-conjugated alkaline phosphatase) prepared in 5% skim milk blocking buffer (1:10,000) at 37 °C for 1 h. Then the membrane was washed with PBST buffer four times. The positive bands on the membrane were detected using an ultrasensitive ECL chemiluminescence kit (Sangon Biotech, Shanghai, China).

### 2.7. Cleavage of SUMO Fusions

The fusion peptide was reacted with 1 U of SUMO protease per 50 μg peptide in a buffer containing 20 mM Tris–HCl and 5 mM β-mercaptoethanol (pH 8.0) at 25 °C for 1 h. After the fusion peptide was cleaved by the SUMO protease, the sample was loaded onto a nickel column with Ni-IDA resin. The hepcidin peptide without 6×His and SUMO tags was eluted in the flow-through (unbound) fraction, and the rest was recovered by washing the resin with binding buffer. The purified protein was analyzed on SDS-PAGE to verify its purity.

### 2.8. Antibacterial Activity Assays

#### 2.8.1. Minimum Inhibitory Concentration (MIC)

The MIC of hepcidin peptides (obtained by expression systems and chemical synthesis) was determined against *V. parahaemolyticus*, *E. coli*, and *S. aureus* strains according to the method of Al-Mohammadi et al. (2020) [22]. Bacterial suspensions (10^5^ CFU/mL) were treated with hepcidin peptides at the final concentrations of 1.6, 0.8, 0.4, 0.2, 0.1, 0.05, and 0.025 μmol/mL and shaken for 24 h at 37 °C. The absorbances at 600 nm of the mixtures were determined using a microplate reader (SpectraMax M5, Molecular Devices Co., Ltd., San Jose, CA, USA). The MIC value was defined as the lowest peptide concentration that inhibited the growth of bacteria.

#### 2.8.2. Colony Counting Assay

The antibacterial activity of the purified peptide was compared to that of the synthetic hepcidin peptides. The mature part of crescent sweetlips hepcidin (QSHLSLCRWCCNCCRGNKGCGYCCRF) was synthesized using a solid-phase peptide synthesis method by Nanjing TGpeptide Biotechnology Co., Ltd. (Nanjing, China). An antibacterial activity assay was performed according to the method of Lai et al. (2021) [23]. In brief, several bacterial species, including *V. parahaemolyticus*, *E. coli*, and *S. aureus*, were cultured and used for antibacterial assays. The hepcidin peptide at a concentration of 0.1 μmol/mL was mixed with bacterial suspension (with a concentration of 10^7^ CFU/mL) at a ratio of 1:1 in tubes. The tubes were incubated at 37 °C for 3 h using a rotary shaker with a speed of 160 rpm. After that, the mixtures were taken out and diluted with culture mediums. Then, 100 μL of the samples were spread on an agar plate and then incubated at 37 °C for 24 h. The number of colonies was counted using a Scan 1200 Colony Counter (Interscience Co., Saint Nom, France).

### 2.9. Statistical Analyses

The experimental results were presented as mean ± SD. SPSS statistical software (version 23, IBM Corp., Armonk, NY, USA) was used to analyze the statistically significant differences using one-way analysis of variance (ANOVA) followed by the Duncan test. *p* < 0.05 was considered statistical significance.

## 3. Results

### 3.1. Construction of the Recombinant Plasmid Expressing of Hepcidin Peptide

**Figure 1:**
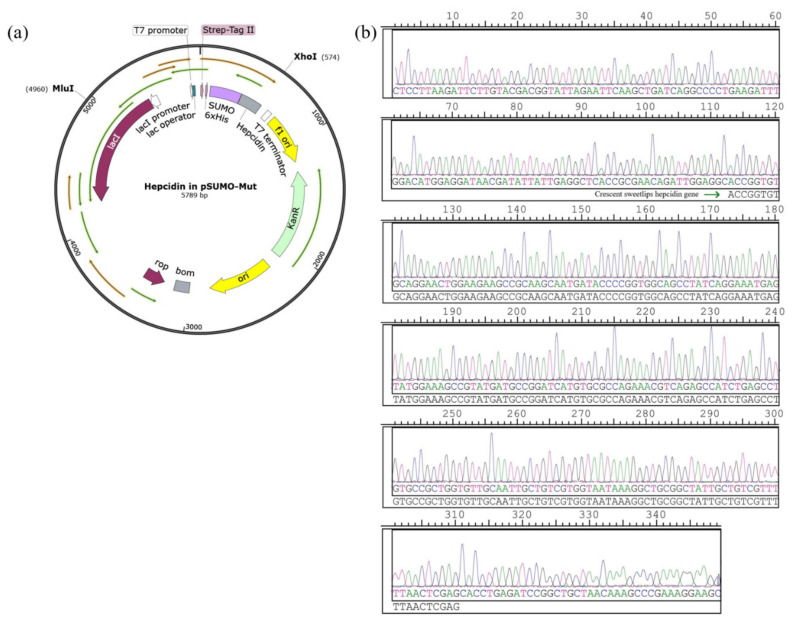
Construction and identification of recombinant plasmid pSUMO-Mut-hepcidin. (**a**) Schematic representation of recombinant plasmid. (**b**) DNA sequencing results. The peaks of nucleobases A, G, C, and T were indicated by green, black, blue, and pink, respectively.

The synthetic hepcidin gene was ligated with pSUMO-Mut to construct the expression plasmid pSUMO-Mut-hepcidin (Figure 1a). The obtained recombinant plasmid was sequenced using the primer GGCCCCAAGGGGTTATGCTAGT. As shown in Figure 1b, the DNA sequencing result confirmed that the inserted DNA fragment was matched to the nucleotide sequence of mature crescent sweetlips hepcidin peptide, demonstrating that the expression plasmid encoding His-SUMO-tagged crescent sweetlips hepcidin peptide was successfully constructed.

### 3.2. Effect of Culture Mediums on the Expression of Recombinant Hepcidin Peptide

**Figure 2:**
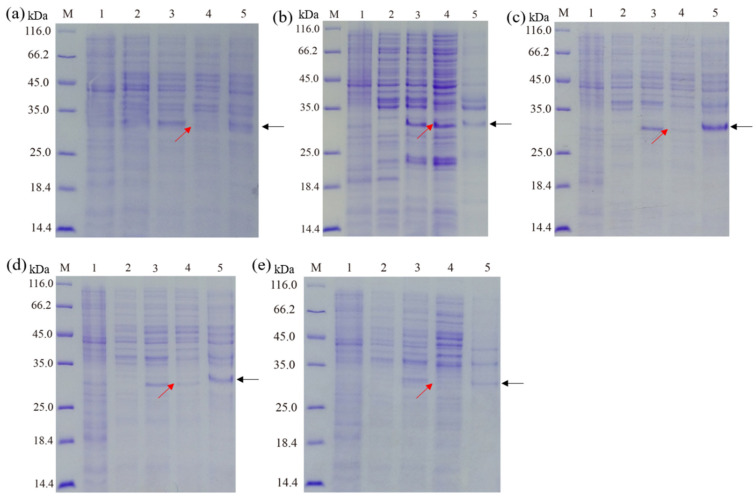
Media optimization for expression of recombinant proteins. (**a**) LB. (**b**) TB. (**c**) SOC. (**d**) SOB. (**e**) 2× YT. Lane M, marker; Lane 1, pSUMO-Mut vector; Lane 2, un-induced culture; Lane 3, induced culture; Lane 4, supernatant of the bacteria lysate; Lane 5, sediment of the bacteria lysate. The recombinant proteins in the supernatant and sediment of the bacteria lysate are indicated by the red and black arrows, respectively.

The effects of culture mediums (LB, TB, SOC, SOB, and 2× YT) on the expression of fusion hepcidin peptides in *E. coli* are shown in Figure 2. Although the bacteria cultured in all tested culture mediums were found to be capable of producing recombinant hepcidin peptides upon IPTG induction, it is clear that the highest level of soluble recombinant hepcidin peptides (in the supernatant of lysate, lane 4) was observed in *E. coli* cultured in the TB medium. In contrast, the recombinant hepcidin peptide was mainly expressed as an inclusion body (lane 5) using LB, SOB, SOC, and 2× YT mediums. These results suggested that the TB medium might be optimal for the expression of the fusion hepcidin peptide in *E. coli*. Indeed, several previous studies also reported that various recombinant proteins could be effectively expressed in *E. coli* using a TB medium [24]. The reason might be that the composition of the TB medium is more favorable for soluble expression than other mediums [25].

### 3.3. Effect of IPTG Concentration, Induction Time, and Induction Temperature on the Expression of Recombinant Hepcidin Peptide

**Figure 3:**
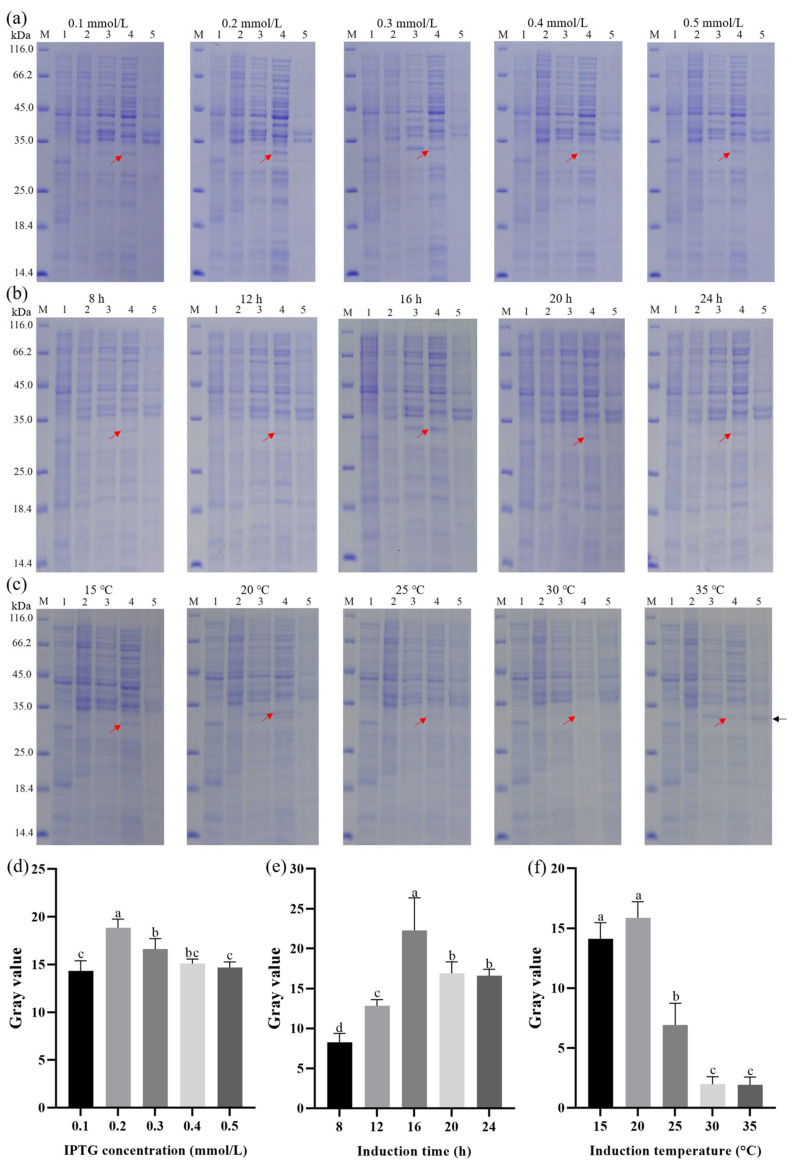
Effect of induction conditions on the expression of recombinant proteins (**a**) IPTG concentration. (**b**) Induction time. (**c**) Induction temperature. (**d**) Gray value of recombinant protein under different IPTG concentrations. (**e**) Gray value of recombinant protein under different induction times (**f**) Gray value of recombinant protein under different induction temperatures. Lane M, marker; Lane 1, pSUMO-Mut vector; Lane 2, un-induced culture; Lane 3, induced culture; Lane 4, supernatant of the bacteria lysate; Lane 5, sediment of the bacteria lysate. The recombinant proteins in the supernatant and sediment of the bacteria lysate are indicated by the red and black arrows, respectively. Different lowercase letters indicate significant differences among different treatments.

Results presented in Figure 3a showed the influence of IPTG concentration on the expression of His-SUMO-hepcidin peptide in *E. coli* Arctic Express (DE3). The SDS-PAGE results showed that all IPTG concentrations produced soluble His-SUMO-hepcidin. Notably, it was also observed that at the IPTG concentration of 0.2 mM, the band corresponding to His-SUMO-hepcidin showed the highest density among all tested IPTG concentrations.

Similarly, the effect of induction time on the expression of the fusion peptide was also explored. As shown in Figure 3b, the His-SUMO-hepcidin peptide could be found in the supernatant of bacterial lysate at different culture times (8, 12, 16, 20, and 24 h) after induction with IPTG (0.2 mM). The strongest band of recombinant peptide was observed in the lysate of *E. coli* cells harvested at 16 h post-induction.

In addition, the effect of induction temperature (15, 20, 25, 30, 35 °C) on the expression of soluble His-SUMO-hepcidin was next determined. As shown in Figure 3c, the highest level of soluble His-SUMO-hepcidin was obtained from bacterial lysate with an induction temperature of 20 °C. Notably, a sharp decrease in soluble His-SUMO-hepcidin expression was observed when the induction temperature reached 25 °C, indicating a higher induction temperature (>25 °C) resulted in rapid accumulation of inclusion bodies.

### 3.4. Optimization of Induction Conditions by RSM

Regression analysis for linear, interaction, and quadratic terms was performed, and the results are listed in Table S2. A low value of the coefficient of variation (3.28%) clearly illustrated a high degree of precision and a good deal of reliability in the experiment values. The model was significant (*p* < 0.0001), while the lack of fit was not statistically significant (*p* = 0.1645), which demonstrated that the model could be used to guide the optimization [26]. The coefficient of determination (R^2^) was found to be 0.9906, which implied a good fitness of the model which can explain 99.06% of the variability in the response [27]. The model had a high adjusted coefficient of determination (adjusted R^2^ = 0.9785), which also confirmed that the model was highly significant. Among the three factors, induction temperature had the greatest significant effect on the gray value (*p* < 0.0001), followed by IPTG concentration (*p* = 0.0495). However, induction time was not significant (*p* = 0.3293). This result indicated that induction temperature and IPTG concentration had a significantly greater effect on the gray value compared to induction time. The effects of the three quadratic terms A2, B2, and C2 were all significant (*p* < 0.0001), and this result illustrated that they were the significant factors influencing the response. The interaction terms AB and AC were significant (*p* < 0.05), and this illustrated that the interaction between two independent variables (IPTG concentrations and induction time; IPTG concentration and induction temperature) could significantly affect the response value. Taken together, the simplified second-order polynomial equation for gray value (Y) in terms of the original variables was expressed as follows:

(1) $$Y=-145.0905+163.4425A+9.48587B+8.44945C+2.0875\mathrm{AB}-1.655\mathrm{AC}-0.0385\mathrm{BC}-395.075A^{2}\;-0.28739B^{2}-0.19903C^{2}\;\;\;\;$$

where A, B, and C are IPTG concentration, induction time, and induction temperature, respectively.

The fitted polynomial equation was graphically represented as a three-dimensional response surface and two-dimensional contour plots to visualize the relationship between the experimental levels of variables and response (Figure S1a–f). Figure S1a showed the relative effects of IPTG concentration and induction time on gray value, while the other factor was held at the zero-coded level (the center value of the testing ranges). We found that there was an increasing trend in the gray value accompanying the increases in IPTG concentration and induction time, and the maximum gray value was obtained when the IPTG concentration was about 0.21 mmol/L and the induction time was about 16 h. The effects of IPTG concentration and induction temperature were shown in the three-dimensional response surface plot (Figure S1b), where the induction time was constant at the 0 level. Based on the graph, variation in IPTG concentration and induction temperature revealed a quadratic effect on the gray value. The gray value increased as IPTG concentration and induction temperature increased, but beyond IPTG concentration of 0.21 mmol/L and induction temperature of 19 °C, the gray value began to decline. Based on Figure S1c, the significant effect of induction temperature can be easily observed, where the relatively lower induction temperature enhanced the soluble expression of His-SUMO-hepcidin and, contrary to this, the higher temperature reduced the desired expression of His-SUMO-hepcidin. However, an induction temperature below 18 °C would also slightly reduce the production of SUMO-hepcidin. The highest gray value occurred within the induction time of 16.2~16.4 h and the induction temperature of 18.6~19.1 °C. The shape of the two-dimensional contour plot reflected the intensity of the interaction between the two variables, and the ellipse showed a stronger interaction effect than that of the circle [28]. As shown in Figure S1d–f, the elliptical shapes of the contour plot indicated that the interactions between IPTG concentration and induction time and induction temperature were significant. These results were also proven by the *p* value, as shown in Table 1.

In conclusion, optimal induction conditions for His-SUMO-hepcidin were: IPTG concentration 0.21 mmol/L, induction time 16.01 h, and induction temperature 18.81 °C, based on the results of RSM. When the experiment was carried out at optimal conditions, the gray value of His-SUMO-hepcidin on SDS-PAGE was determined to be 27.81 ± 2.32, which was similar to its predicted value (27.42).

### 3.5. Purification of Recombinant Peptide

**Figure 4:**
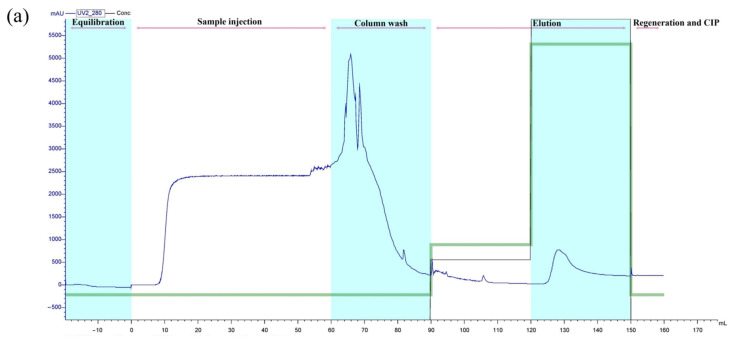

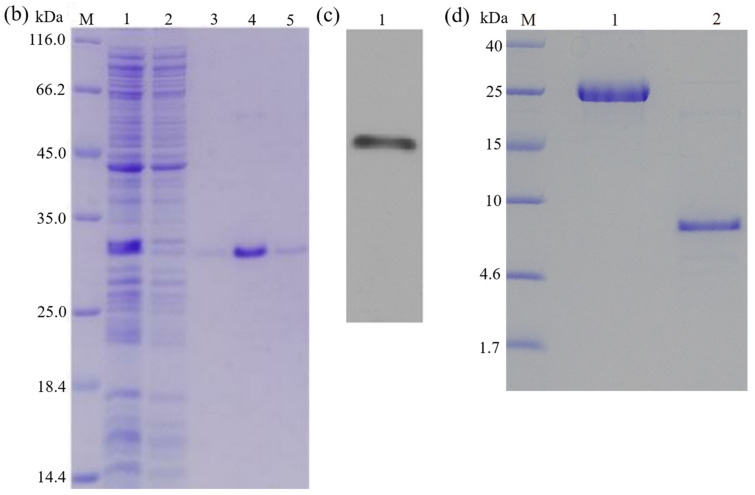
Purification of recombinant soluble hepcidin peptide. (**a**) Elution profile of the fusion protein using affinity chromatography on an Ni-NTA resin. The blue curve indicates the changes of absorbance value at 280 nm; The green curve indicates the changes of imidazole concentrations in the buffer. (**b**) SDS–PAGE analysis of purified fusion proteins. Lane M, marker; Lane 1, supernatant of the bacteria lysate; Lane 2, flow through; Lanes 3–5, purified fusion protein. (**c**) Specificity determination by Western blotting. Lane 1, purified fusion protein. (**d**) The hepcidin peptide obtained after cleaving by SUMO protease and purifying by Ni-IDA column. Lane M, marker; Lane 1, His-SUMO tag; Lane 2, hepcidin peptides.

As seen in Figure 4a, elution of the Ni-NTA resin yielded a sharp peak eluting from 124 to 144 mL, which contained His-SUMO-hepcidin. The purified recombinant peptide was further identified using SDS-PAGE, on which only a single band at approximately 31 kDa appeared (Figure 4b). This was consistent with the predicted molecular mass of the target peptide. The western blot analysis using the anti-6×His tag monoclonal antibody further confirmed that the recombinant His-SUMO-hepcidin peptide was successfully expressed by *E. coil* (Figure 4c).

### 3.6. Cleavage of the Fusion Tag

After cleaving by the SUMO protease and purifying by a nickel column, the obtained peptide was analyzed on SDS–PAGE again. As shown in Figure 4d, a single band with a molecular weight of 7.2 kDa appeared, which corresponded to the theoretical molecular weight of crescent sweetlips hepcidin predicted by the web-based software (http://web.expasy.org/compute.pi (accessed on 25 June 2023)). The result indicated that the 6×His and SUMO tags were successfully removed from the fusion peptide.

### 3.7. Antibacterial Activity of Recombinant Protein

#### 3.7.1. MIC

As shown in Figure S2, the MICs of purified prokaryotic expressed hepcidin peptide against *V. parahaemolyticus*, *E. coli*, and *S. aureus* were 0.1, 0.2, and 0.2 μmol/mL, respectively. Meanwhile, MIC values were 0.1, 0.2, and 0.2 μmol/mL for synthetic hepcidin peptide as determined against *V. parahaemolyticus*, *E. coli*, and *S. aureus*. The results indicated that the antibacterial activities of crescent sweetlips hepcidin peptides prepared by both methods were comparable.

#### 3.7.2. Colony Counting Assay

**Figure 5:**
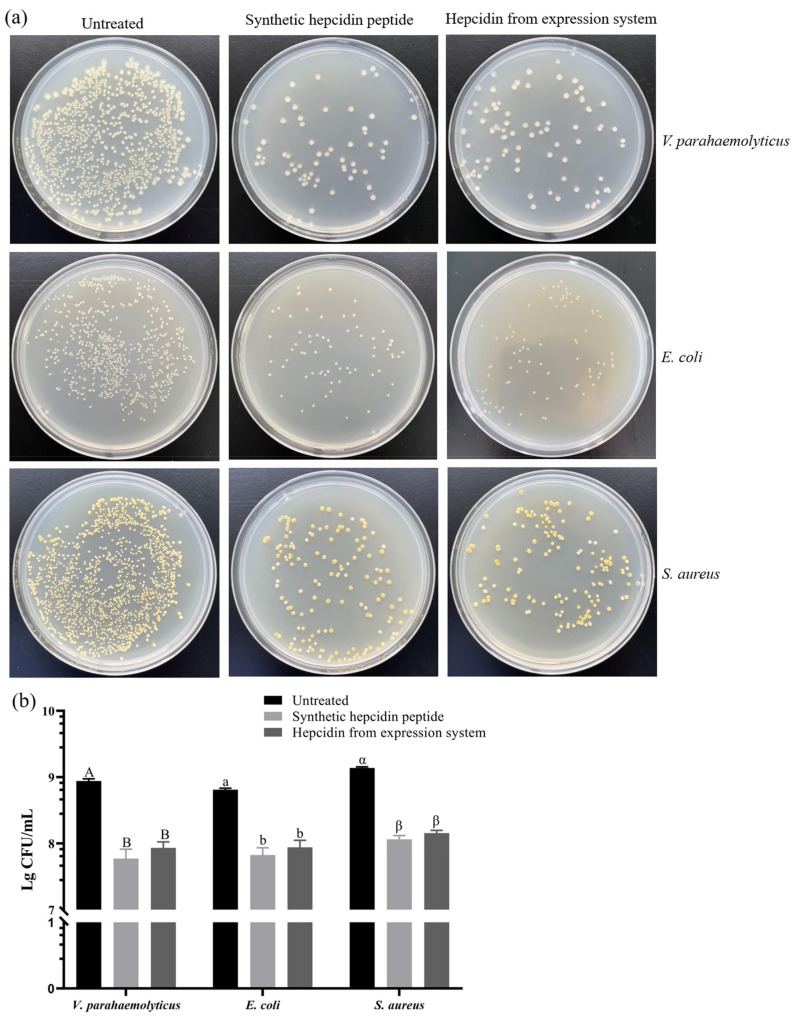
Antibacterial activities of hepcidin peptide prepared by different methods. (**a**) The photographs of bacteria colonies (diluted by 10^5^ times; 100 μL). (**b**) The number of bacteria colonies. Different capital letters, lowercase letters, and Greek letters indicate statistically significant differences in the colony numbers of *V. parahaemolyticus*, *E. coli*, and *S. aureus* treated with different samples, respectively.

Next, the antibacterial activity of prokaryotic-expressed crescent sweetlips hepcidin peptide against *V. parahaemolyticus*, *E. coli*, and *S. aureus* was compared with its chemically synthesized counterpart. As shown in Figure 5, purified peptide at 0.02 mmol/mL led to 90.07%, 86.27%, and 89.55% reductions in bacterial colonies of *V. parahaemolyticus*, *E. coli*, and *S. aureus*, respectively. Similarly, the chemically synthesized crescent sweetlips hepcidin peptide resulted in comparable decreases in the colony numbers of *V. parahaemolyticus*, *E. coli*, and *S. aureus* by 93%, 89.46%, and 91.54%, respectively. Notably, our results also suggested that crescent sweetlips hepcidin showed higher bactericidal activity against *V. parahaemolyticus* than *E. coli* and *S. aureus*. This result may be caused by the fact that the cell membranes of *Vibrio parahaemolyticus* are more sensitive to antimicrobial peptides than those of other tested bacteria, as antimicrobial peptides have been shown to inactivate bacteria by destroying membranes [29].

## 4. Discussion

For peptide production, chemical synthesis and prokaryotic/eukaryotic expression systems are currently the most widely adopted strategies. Chemical synthesis using the condensation reaction has rapidly progressed in the last decades and could be used to produce a range of functional peptides with high purity, good solubility, and high activity [30]. However, a number of disadvantages, such as the usage of hazardous solvents and reagents (e.g., trifluoroacetic acid) during the synthetic and purification processes, multiple reaction steps and inevitable byproducts, and a high purification cost (especially for longer peptides), often make chemical synthesis of antibacterial peptides beyond reasonable expenses for its industrial applications [31]. In contrast, recombinant production of antibacterial peptides in *E. coli* using cleavable tags is defined as a simple, inexpensive production process with the advantages of quick expression and high product yields [32]. Therefore, it seems that the proposed method for industrialized production of hepcidin is its recombinant expression.

Hepcidin is a kind of antibacterial peptide with great potential in aquaculture and food preservation [33]. Indeed, several studies have reported the eukaryotic expression of recombinant fish hepcidin [16]. For example, Ma et al. (2018) [34] showed that the olive flounder hepcidin gene was inserted into the pPIC9K vector and successfully expressed by the Pichia pastoris expression system. The obtained recombinant peptide exhibited desirable antibacterial activities against *Streptococcus iniae* and *Edwardsiella tarda*. Similarly, recombinant tilapia hepcidin was also successfully produced by a *Pichia pastoris* expression system and exhibited broad-spectrum antimicrobial activity [35]. However, several studies also demonstrated that recombinant hepcidin peptides expressed in the *E. coli* expression system were prone to aggregate and form inclusion bodies [36].

In the present study, the hepcidin gene was successfully cloned into the pSUMO-Mut vector and transformed into *E. coli* Arctic Express (DE3). Indeed, the application of the SUMO fusion strategy to express antimicrobial peptides has been widely reported [37], which significantly improves the peptide’s correct folding through its chaperone properties [32]. Furthermore, the chaperoning properties of the SUMO tag also facilitate proper peptide folding, which enhances the correct folding and biological activity of the recombinant peptides [38].

Optimal medium composition is a crucial aspect of the successful expression of soluble proteins in *E. coli* [39]. In the present study, five different mediums (LB, TB, SOC, SOB, and 2× YT supplemented with kanamycin) were tested for *Escherichia coli* Arctic Express (DE3) expressing His-SUMO-hepcidin. Results: Bacteria cultured in TB medium resulted in the highest expression of soluble His-SUMO-hepcidin and the lowest level of inclusion body compared to other tested mediums. Indeed, the TB medium is a nutritionally rich medium that has been widely used for the expression of recombinant proteins in *E. coli*. Previous studies have suggested that compared with other mediums, the TB medium possesses a number of advantages, such as good buffering capacity to maintain a desirable pH value during bacteria growth and the presence of glycerol as an additional carbon source [25,40]. Hence, TB medium was selected in the present study in order to enhance the expression of soluble His-SUMO-hepcidin in *E. coli*.

It has been demonstrated that induction conditions play a key role in prokaryotic expression [41]. Particularly, optimization of IPTG concentration, induction time, and induction temperature were prominent approaches to enhancing recombinant protein production. Therefore, these three factors were selected to be optimized using RSM, an effective tool for optimizing the process when several factors and interactions affect the desired response [42], in the current study. Indeed, RSM and Box-Behnken design have been successfully used for the optimization of induction conditions to maximize the protein yield. The obtained results showed that induction temperature profoundly influences peptide yield compared to IPTG concentration and induction time. Induction temperature has been widely reported previously as a key factor for enhancing the correct folding of target proteins in recombinant *E. coli* and avoiding the formation of inclusion bodies. For instance, Hadj Sassi et al. (2017) found that induction at low temperatures leads to an increase in the amount of soluble recombinant protein pectin lyase with less inclusion in body formation [43].

Ni-IDA affinity chromatography is one of the most commonly used methods to purify His-tagged recombinant proteins expressed in *E. coli* [44]. The His-tagged recombinant proteins can be immobilized by metal ions (e.g., Ni^2+^ or Cu^2+^) in the affinity chromatography column and eluted by imidazole solution [45]. In the present study, a single peak was observed after eluting from the Ni–NTA column, indicating that the obtained peptide possessed high purity. Subsequently, the His-SUMO-hepcidin fusion protein was cleaved by SUMO protease, and its purity was also verified by SDS-PAGE, with only a single band appearing.

Furthermore, the antibacterial activity of the purified hepcidin peptide was detected in *V. parahaemolyticus*, *E. coli*, and *S. aureus*. Results showed that prokaryotic-expressed hepcidin peptides could significantly inhibit the growth of all three tested bacteria and possessed similar antibacterial activities to the chemically synthesized hepcidin peptides. These results confirmed that prokaryotic (*E. coli*) expression could be a feasible way to produce the hepcidin peptide of crescent sweetlips with desirable anti-bacterial activity.

## 5. Conclusions

In the current study, we described a prokaryotic expression approach to produce soluble, active, and purified crescent sweetlips hepcidin peptide using *E. coli* Arctic Express (DE3). Our findings suggest that the prokaryotic expressed crescent sweetlips hepcidin peptide under optimal induction conditions possessed desirable antimicrobial activity against both Gram-negative and Gram-positive bacteria. Taken together, the results of the present study may assure further investigation toward industrialized production of antimicrobial crescent sweetlips hepcidin peptide.

## Figures and Tables

**Table 1:** Analysis of variance (ANOVA) for the response surface quadratic model used for the optimization of induction conditions.

Source	Sum of Squares	df	Mean Square	F Value	*p*-Value Prob > F	Significance
Model	342.19	9	38.02	81.93	<0.0001	**
A	2.61	1	2.61	5.63	0.0495	*
B	0.51	1	0.51	1.10	0.3293	
C	42.09	1	42.09	90.70	<0.0001	**
AB	2.79	1	2.79	6.01	0.0440	*
AC	2.74	1	2.74	5.90	0.0455	*
BC	2.37	1	2.37	5.11	0.0583	
A^2^	65.72	1	65.72	141.61	<0.0001	**
B^2^	89.03	1	89.03	191.83	<0.0001	**
C^2^	104.24	1	104.24	224.62	<0.0001	**
Residual	3.25	7	0.46			
Lack of Fit	2.23	3	0.74	2.91	0.1645	not significant
Pure Error	1.02	4	0.26			
Cor Total	345.44	16				

* *p* < 0.05, significant difference; ** *p* < 0.01, highly significant difference.

## Data Availability

All data generated or analyzed during this study are included in this published article and its additional file.

## Supplementary Materials

The following supporting information can be downloaded at: https://www.mdpi.com/article/10.3390/cimb45090456/s1. Table S1: Factors and levels in the RSM experimental design; Table S2: Experimental design used in RSM studies and responses for the optimization of induction conditions. Figure S1: The three-dimensional response surface plots and two-dimensional contour plots of the relative effects on gray value. Figure S2: The MICs of crescent sweetlips hepcidin peptides prepared by different methods.
