# Supplementary material for: Prokaryotic Expression, Purification, and Antibacterial Activity of the Hepcidin Peptide of Crescent Sweetlips (Plectorhinchus cinctus)
Source: Curr Issues Mol Biol. 2023 Aug 31;45(9):7212–27. doi: 10.3390/cimb45090456 (PMC10528233; doi:10.3390/cimb45090456)

**Table S1.** Factors and levels in the RSM experimental design.

| Factors | Code | Code Level | | |
|---|---|---|---|---|
| | | **−1** | **0** | **1** |
| IPTG concentrations (mmol/L) | A | 0.1 | 0.2 | 0.3 |
| Induction time (h) | B | 12 | 16 | 20 |
| Induction temperatures (°C) | C | 15 | 20 | 25 |

**Table S2.** Experimental design used in RSM studies and responses for the optimization of induction conditions.

| Run Number | Coded Variables | | | Gray Values |
|---|---|---|---|---|
| | **A** | **B** | **C** | **Y** |
| 1 | 0 | 0 | 0 | 27.38 |
| 2 | 1 | -1 | 0 | 19.15 |
| 3 | 0 | 0 | 0 | 26.22 |
| 4 | -1 | -1 | 0 | 19.51 |
| 5 | -1 | 1 | 0 | 16.31 |
| 6 | 1 | 1 | 0 | 19.29 |
| 7 | 0 | 0 | 0 | 27.37 |
| 8 | 1 | 0 | 1 | 15.46 |
| 9 | 0 | 0 | 0 | 27.39 |
| 10 | 0 | 0 | 0 | 27.21 |
| 11 | 1 | 0 | -1 | 21.89 |
| 12 | 0 | 1 | -1 | 20.77 |
| 13 | -1 | 0 | -1 | 19.26 |
| 14 | 0 | 1 | 1 | 14.83 |
| 15 | 0 | -1 | 1 | 15.85 |
| 16 | 0 | -1 | -1 | 18.71 |
| 17 | -1 | 0 | 1 | 16.14 |

**Figure S1 The three-dimensional response surface plots and two-dimensional contour plots of the relative effects on gray value.** (**a**) and (**d**): IPTG concentration and induction time; (**b**) and (**e**): IPTG concentration and induction temperature; (**c**) and (**f**): Induction temperature and induction time.

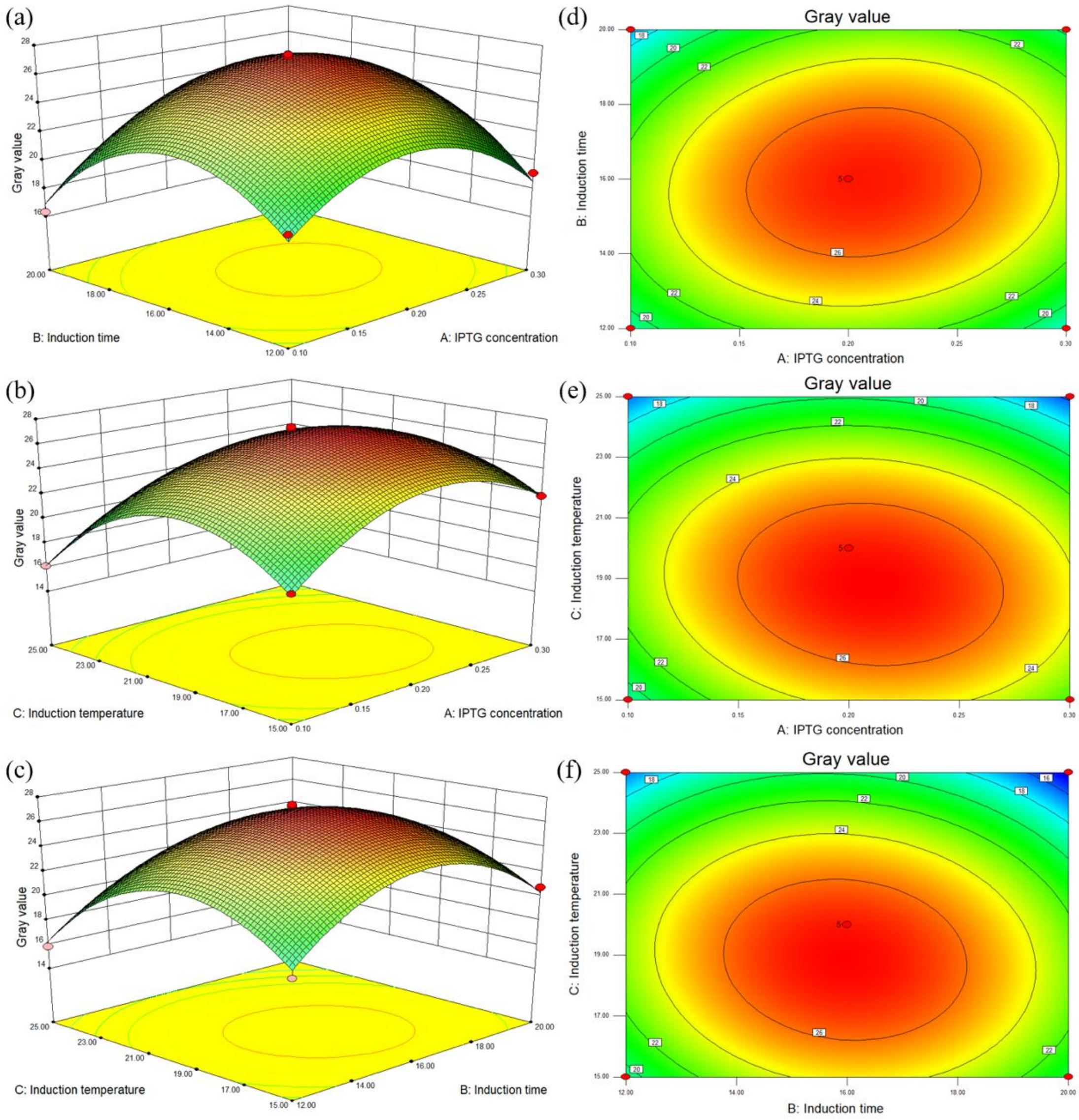

**Figure S2 The MICs of crescent sweetlips hepcidin peptides prepared by different methods.** (**a-c**) The MIC of synthetic hepcidin peptide against *V. parahaemolyticus*, *E. coli*, and *S. aureus*. (**d-f**) The MIC of hepcidin peptide obtained by expression system against *V. parahaemolyticus*, *E. coli*, and *S. aureus*. * $p < 0.05$, compared with 0 h.

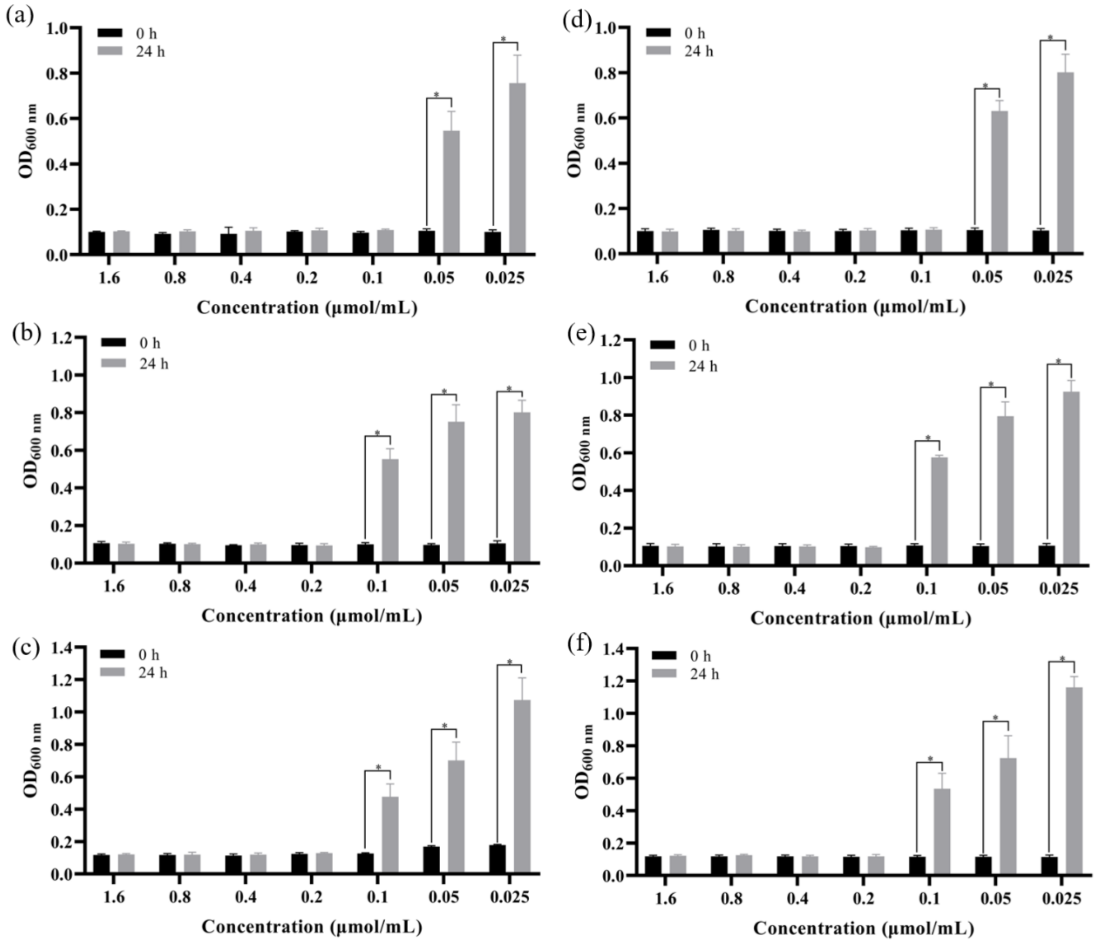

Supplement: Supplementary file 1 [file cimb-45-00456-s001.zip › cimb-2566401-supplementary.pdf]
